# Adolescent Coordinated Transition (ACT) to improve health outcomes among young people living with HIV in Nigeria: study protocol for a randomized controlled trial

**DOI:** 10.1186/s13063-017-2347-z

**Published:** 2017-12-14

**Authors:** Nadia A. Sam-Agudu, Jennifer R. Pharr, Tamara Bruno, Chad L. Cross, Llewellyn J. Cornelius, Prosper Okonkwo, Bolanle Oyeledun, Hadiza Khamofu, Ayodotun Olutola, Salome Erekaha, William Nii Ayitey Menson, Echezona E. Ezeanolue

**Affiliations:** 1 Pediatric and Adolescent HIV Unit, Clinical Services, and International Research Center of Excellence, Institute of Human Virology Nigeria, Abuja, Nigeria; 2 Division of Epidemiology and Prevention, Institute of Human Virology, University of Maryland School of Medicine, Baltimore, OH USA; 3 Global Health Initiative, School of Community Health Sciences, University of Nevada Las Vegas, 4505 S. Maryland Parkway, Las Vegas, NV 89154 USA; 4 School of Medicine and School of Community Health Sciences, University of Nevada, Las Vegas, NV USA; 5 School of Social Work and College of Public Health, University of Georgia Athens, Athens, GA USA; 6 APIN Public Health Initiatives, Abuja, Nigeria; 7 Centre for Integrated Health Programs, Abuja, Nigeria; 8 FHI 360, Abuja, Nigeria; 9 Center for Clinical Care and Clinical Research Nigeria, Abuja, Nigeria

**Keywords:** HIV, Healthcare transition, Adolescent, Retention, Viral suppression, Mental health, Nigeria

## Abstract

**Background:**

Adolescents living with HIV (ALHIV) have worse health outcomes than other populations of people living with HIV. Contributing factors include lack of standard and comprehensive procedures for ALHIV transitioning from pediatric to adult care. This has contributed to poor retention at, and following transition, which is problematic especially in high ALHIV-burden, resource-limited settings like Nigeria.

**Methods:**

Using a two-arm cluster randomized control design, the Adolescent Coordinated Transition (ACT) trial will measure the comparative effectiveness of a graduated transition and organized support group intervention against the usual practice of abrupt transfer of Nigerian ALHIV from pediatric to adult care. This study will be conducted at 12 secondary and tertiary healthcare facilities (six intervention, six control) across all six of Nigeria’s geopolitical zones. The study population is 13- to 17-year-old ALHIV (*N* = 216, *n* = 108 per study arm) on antiretroviral therapy. Study participants will be followed through a 12-month pre-transfer/transition period and for an additional 24 months post transfer/transition. The primary outcome measure is the proportion of ALHIV retained in care at 12 and 24 months post transfer. Secondary outcome measures are proportions of ALHIV achieving viral suppression and demonstrating increased psychosocial wellbeing and self-efficacy measured by psychometric tests including health locus of control, functional social support, perceived mental health, and sexual risk and behavior.

**Discussion:**

We hypothesize that the ACT intervention will significantly increase psychosocial wellbeing, retention in care and ultimately viral suppression among ALHIV. ACT’s findings have the potential to facilitate the development of standard guidelines for transitioning ALHIV and improving health outcomes in this population. The engagement of a consortium of local implementing partners under the Nigeria Implementation Science Alliance allows for nationwide study implementation and expedient results dissemination to program managers and policy-makers. Ultimately, ACT may also provide evidence to inform transitioning guidelines not only for ALHIV but for adolescents living with other chronic diseases in resource-limited settings.

**Trial registration:**

ClinicalTrials.gov, ID: NCT03152006. Registered on May 12, 2017.

**Electronic supplementary material:**

The online version of this article (doi:10.1186/s13063-017-2347-z) contains supplementary material, which is available to authorized users.

## Background

Between 2005 and 2012, global acquired immunodeficiency syndrome (AIDS)-related deaths fell by 30% among people living with human immunodeficiency virus (HIV); however, mortality among adolescents living with HIV (ALHIV) increased by 50% in the same period [1]. Nearly 92% of these deaths occurred in sub-Saharan Africa, where AIDS is the leading cause of mortality among adolescents [2–4]. Of the estimated 1.75 million ALHIV in sub-Saharan Africa, approximately 240,000 are living in Nigeria, which represents the second highest burden of adolescent HIV globally [2]. In 2013, an estimated 11,000 deaths occurred among Nigerian ALHIV, representing 9.2% of AIDS-related deaths among all African adolescents [2].

Loss to follow-up (LTFU) and poor retention in care are associated with increased risk of mortality and morbidity among ALHIV [5, 6]. Studies have demonstrated that LTFU rates are higher and retention lower among ALHIV, compared to adult patients [6, 7]. A recent nationwide study reported a LTFU rate among Nigerian ALHIV at 16.1% after a mean follow-up of 28 months [8]. In addition to LTFU, poor adherence to antiretroviral therapy (ART) results in virologic treatment failure and contributes to increased mortality among adolescents [9–11].

The healthcare transition period is particularly high risk for LTFU and other poor health outcomes among ALHIV. Healthcare transition is defined as “the purposeful, planned movement of adolescents and young adults with chronic physical and medical conditions from child-centered to adult-oriented healthcare systems” [12]. Barriers to successful transition have been categorized as: (1) patient/family related (e.g., knowledge deficits in self-management skills); (2) provider-related (e.g., lack of time for transitioning, confusion about roles, and pediatricians’ belief that adult providers would provide inadequate care); and (3) system-related (e.g., lack of formal transitioning program, lack of trained adult providers to competently care for transferred adolescents, and challenges in establishing an interdisciplinary team) [13].

A recent nationwide survey conducted by our study group determined that Nigerian ALHIV are routinely transferred from pediatric to adult care at age 15 or 18 years [14]. Factors limiting successful transition and post-transition retention include: lack of preparation, poor logistics, poor communication between pediatric and adult providers, loss of the “pediatric family” relationships of ALHIV, transition readiness and system-related issues such as access to care, and continuity of care post transition [15].

The most commonly identified components of successful transition include: provision of psychological support to help adolescents deal with anxieties and anger, provision of information, e.g., on risky sexual behavior and access to/navigation of medical services, and providing education on disease state and the importance of adherence to treatment [16–21]. Nigerian ALHIV are also reported to seek social support as a coping strategy for stress related to visiting the hospital and taking drugs regularly [22]. Additional factors that promote successful healthcare transition for ALHIV include: open communication between pediatric and adult staff, peer educator support, access to tailored HIV information, and capacity-building for ALHIV to meet their own psychosocial needs especially in self-efficacy and self-esteem [4, 16–20].

While national policy documents in Nigeria recognize HIV among adolescents as an important issue, none of them address healthcare transition and no guidelines exist for transition of Nigerian adolescents living with HIV or, for that matter, any chronic health condition [23, 24]. Therefore, there is an urgent need to develop a feasible, acceptable, and sustainable model of healthcare transitioning for adolescents in Nigeria. Nigeria’s healthcare system remains weak, and HIV care is predominantly foreign donor-supported [25]. Any new intervention or program should require relatively little additional external funding. Thus, the intervention we propose, Adolescent Coordinated Transition (ACT), is designed to integrate well with, and exert leverage on, the existing healthcare system whilst incorporating effective strategies for healthcare transition.

### Conceptual framework

**Fig. 1:**
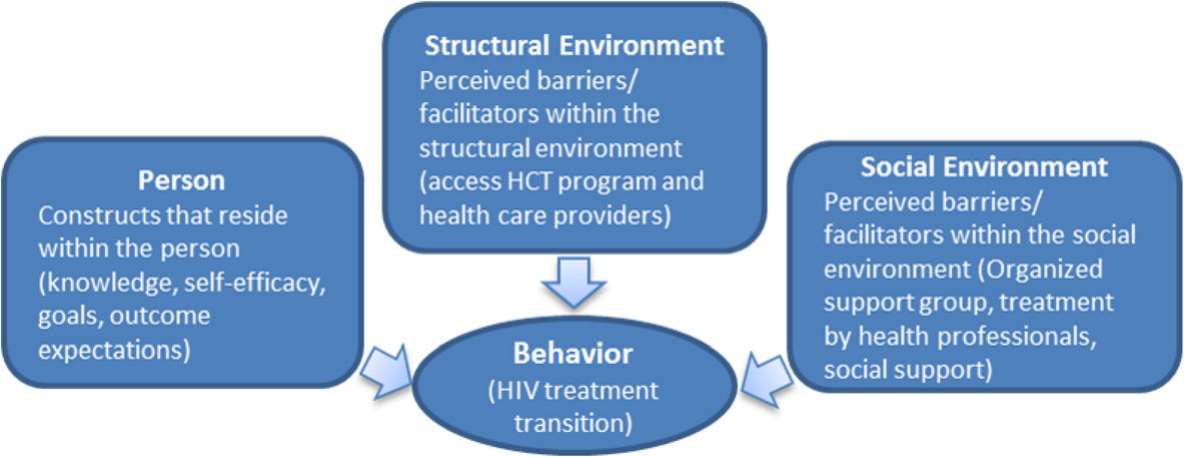
Overview of the social cognitive theory

The ACT intervention relies on the social cognitive theory (SCT) (Fig. 1). Although we considered other frameworks such as the Health Belief Model and the Ecological Model, the constructs of the SCT are particularly relevant to HIV/AIDS treatment and risk reduction among adolescents. Many identified challenges lie with the adolescent or within their environment. SCT recognizes that health behavior is influenced by factors within the person (knowledge of the importance of HIV treatment, how to access treatment during transition, and self-efficacy for service utilization in adult clinics) as well as facilitators or barriers within a person’s social and structural environment (the HIV clinic, peers, or support groups).

### Study objectives and hypotheses

This study’s primary objective is to measure the comparative effectiveness of the ACT intervention versus usual care on post-transfer retention in care among ALHIV at 12 and 24 months post transfer. The ACT intervention encompasses a graduated transition period coordinated with an organized support group, which aims to increase retention rates among ALHIV after transfer to adult care. The coordination denotes a complementary pediatric-adult team approach to support the clinical and psychosocial health of the transitioning ALHIV. The graduated transition provides an extended period of time for the transitioning ALHIV to become familiar with adult care while separating from pediatric care. A transition period, rather than abrupt transfer, minimizes the grief associated with the loss of the pediatric “family” and maximizes opportunities for the ALHIV and adult care providers to establish trusting relationships. The ACT intervention is to be compared to the usual care which is abrupt transfer of ALHIV to adult care with neither a transition period nor an organized support group.

ACT’s secondary objectives are:

- To measure the comparative effectiveness of the ACT intervention versus usual care on post-transfer viral suppression rates among ALHIV at 12 and 24 months post transfer
- To measure the comparative effectiveness of the ACT intervention versus usual care on psychosocial wellbeing as measured by psychometric tests at 12 and 24 months post transfer

We hypothesize that:

1. Compared to those exposed to usual care, ALHIV exposed to the ACT intervention will have significantly higher rates of retention in care
2. Compared to those exposed to usual care, ALHIV exposed to the ACT intervention will have significantly higher rates of viral suppression and higher scores on psychometric testing indicating better psychosocial wellbeing

### Formative consultations

Key information that shaped the design of this study came from multiple formative consultations across the Nigeria Implementation Science Alliance (NISA), a collaboration of the 20 largest HIV implementing partners in Nigeria supported by the President’s Emergency Plan for AIDS Relief (PEPFAR) [26]. NISA-wide consultations held with experienced Nigerian healthcare providers highlighted lack of a transition process for adolescents with common chronic illnesses in Nigeria. There are no guidelines or standard practices for healthcare transitioning for sickle cell anemia, diabetes, asthma, or HIV in Nigeria. As such, the standard of care either is absent or varies widely between providers, healthcare facilities and different parts of the country.

During recent assessments for the establishment of PEPFAR-supported adolescent HIV clinics across NISA’s network, we consulted with Nigerian ALHIV. We found ALHIV eager to be involved in their own healthcare including transitioning; however, they uniformly expressed feeling more comfortable in pediatric care because of time and attention rendered to them. Lessons learned from these consultations (e.g., the need for adult care providers to establish trust with ALHIV, a transition period and not abrupt transfer, and adolescent-friendly psychosocial support) provided critical contextual data to inform our intervention.

## Methods

### Study design

The study is a two-arm, cluster-randomized controlled trial that compares the effectiveness of a graduated, coordinated healthcare transition plus organized support group intervention with the usual care of abrupt transfer among ALHIV. ACT adopts the Type 1 Hybrid Design described by Curran and colleagues, which tests the effect of an intervention on relevant outcomes while observing and gathering information on the implementation of the intervention [27]. The Type 1 Hybrid Design integrates impact and process evaluation with the dual goal of testing intervention effectiveness while identifying factors influencing implementation in context. ACT’s intervention effectiveness will be addressed through measurement and analyses of the primary and secondary objectives. Intervention implementation will be measurement utilizing the RE-AIM framework as a guide for implementation indicators (described in detail below) [28].

### Study setting

The ACT trial is a nationwide study that will be conducted across all six geopolitical zones of Nigeria, namely, the north-west, north-east, north-central, south-west, south-south and south-east zones. Two study sites were selected from each zone to ensure that the intervention is generalizable across the country and can be scaled up expediently if proven effective. The ACT trial is to be implemented by five PEPFAR implementing partners who are NISA members: APIN Public Health Initiatives Limited (APIN), the Center for Clinical Care and Clinical Research Nigeria (CCCRN), Centre for Integrated Health Programs (CIHP), FHI 360 Nigeria, and the Institute of Human Virology Nigeria (IHVN). Collectively, these five organizations support more than 850 comprehensive HIV treatment clinics in multiple settings across Nigeria and provide HIV services to over 24,000 ALHIV of 10 to 19 years old.

### Site selection and randomization

Study sites were selected from among secondary- and tertiary-level PEPFAR-supported healthcare facilities with at least 12 months’ experience in providing comprehensive HIV care and treatment services. Eligible sites were further expected to meet the following criteria:

- At least 20 ALHIV enrolled in care as of 31 July 2016
- Separate pediatric and adult HIV care teams and clinics
- At least one dedicated physician and one dedicated nurse for each pediatric and adult HIV clinic

All sites were assessed using a standardized tool to determine whether they met the selection criteria.

**Fig. 2:**
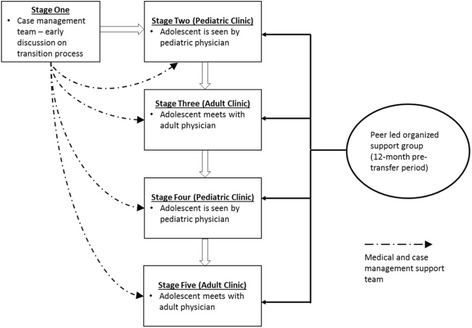
Consolidated Standards of Reporting Trials (CONSORT) flow diagram for Adolescent Coordinated Transition (ACT) trial site selection

Our desire was to meet the suggestions of the Consolidated Standards of Reporting Trials (CONSORT) “extension for cluster designs” checklist in our randomization procedure. To that end, we provide details here of both sequence generation and allocation [29]. Twelve healthcare facilities were selected and randomized 1:1 to the intervention and control arms (Fig. 2). These 12 study sites were pair-matched by geographic location and level of care and then randomly assigned to the intervention group (IG) or to the control group (CG). Particular attention was paid to remove bias of group assignment by concealment at the individual participant level. The randomization of facilities was carried out using scripting language in R (v. 3.3.2) by the project statistician [30]. We first created a random number between 1000 and 10,000 and used the result to seed the group assignment so that the results would be reproducible. Based on the pair-matched locations and level of care, we used the “sample” function to determine which member of each matched facility pair would be assigned to each of CG and IG. Of note, age at transfer for each study site was considered in order to include sites that transferred at 15 years as well as 18 years, according to the results of our survey [14]. All enrolled study participants receiving care at each study site will follow the procedures of their respective randomization arms described below.

### Study population

Study participants will be selected from among ALHIV enrolled in care at participating healthcare facilities.

#### Inclusion and exclusion criteria

Inclusion criteria

1. Documented HIV infection
2. Enrolled in HIV care and taking ART at a designated ACT study site
3. Age 13–17 years: this allows for the following: alignment with target ages for routine transfer for healthcare facilities in Nigeria of 15- and 18-year-olds [14]; the pre-transfer 12-month transition period plus organized support group in the IG while CG is simply observed; and flexibility (albeit limited) in recruitment and study transfer age given the observed flexibility in age at transfer in actual practice
4. Aware of HIV diagnosis

Exclusion criteria

1. Unable or unwilling to provide assent/consent
2. Not medically stable

### Description of the ACT intervention

**Fig. 3:**
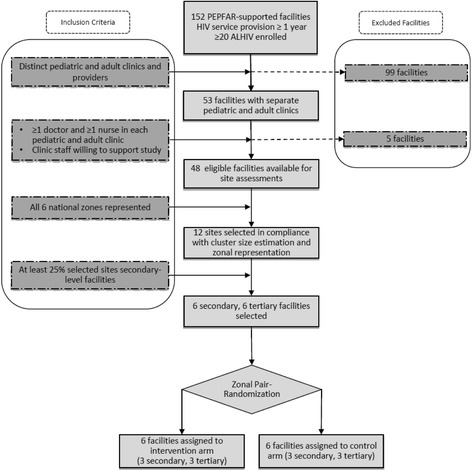
Adolescent Coordinated Transition (ACT) transitioning model showing the 12-month pre-transfer period (adapted from Maturo et al. [19])

The ACT intervention is adapted and modified from the model described by Maturo et al. as shown in Fig. 3 [19]. The model is adapted to national guideline-recommended schedule of clinic visits every 3 months [31]. ACT has three main components:

1. Alternating pediatric-adult visits during the 12-month transition period that allows both adult and pediatric clinicians and the transitioning adolescent to address difficulties related to transition and adapt to the termination of the pediatric provider-patient relationship. Transition period clinic visits will be staggered as a Pediatric-Adult-Pediatric-Adult (PAPA) model with a target of four visits in total
2. A monthly peer-led organized support group (OSG) facilitated by trained young adults living with HIV and guided by a standardized six-module curriculum covering HIV Basics, Treatment and adherence, Support networks, Adolescent rights, Living positively, and Member choice. The OSG curriculum will address topics that will enhance the adolescent’s knowledge of HIV disease and how to self-manage their medical care. Overall, the OSG targets factors that may impact the patient’s transition readiness and psychosocial health measures. ALHIV will have the opportunity to contribute to curriculum content as their OSGs evolve and mature. The OSG will be initiated at enrollment and continued through the 12-month transition period, but to assess potential impact after transfer, it will be extended to the end of the follow-up period at 24 months post transfer
3. A case management team consisting of a physician, a nurse, and a trained patient advocate. Patient advocates already exist in these clinics and are usually HIV-infected adults who are selected based on exemplary treatment adherence and clinic attendance. The patient advocate will accompany study participants to all four PAPA clinic visits and will support the peer facilitator to lead the OSG

In the intervention arm, ALHIV will be transitioned from pediatric to adult care over a period of 1 year. Participants will be enrolled when they are approximately 1 year younger than the routine age of transfer at each study facility (either 15 or 18 years of age). During the 12-month pre-transfer period, ALHIV will be scheduled for clinic visits per the PAPA model. Patient advocates will accompany adolescents to each PAPA clinic visit. In addition, ALHIV receiving care at intervention sites will be encouraged to attend monthly OSG meetings. OSG meetings will be scheduled with routine clinic visits and at adolescent-friendly times to minimize logistical problems and to increase attendance and sustainability. Each OSG will include up to 25 participants.

### Description of the control condition/usual care

The usual “transition” of care for ALHIV in Nigeria is abrupt transfer to adult care. For this protocol, “transfer” is defined as immediate handover of the ALHIV from pediatric to adult care, without a transition period, and no formal or documented communication between pediatric and adult providers prior to, or after transfer. There is also no structured pre-transfer education and/or counseling provided to ALHIV and their caregivers with respect to procedures and expectations in adult care. In CG clinics, ALHIV will stop accessing pediatric care and start accessing the adult HIV clinic at the routine age of transfer at the facility. Adolescents will have access to all available services at the adult clinic including any adult support groups that may be available.

### Participant recruitment

**Fig. 4:**
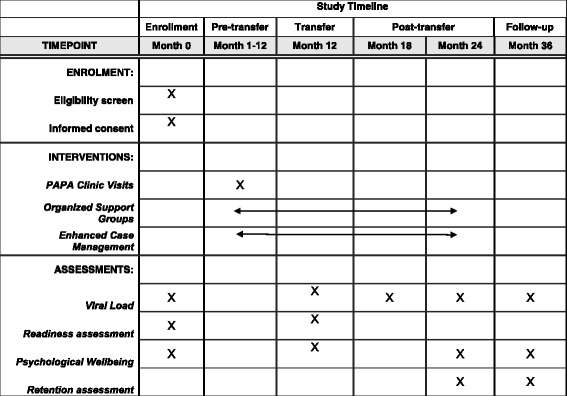
Schedule of enrollment, interventions, and assessments

The Standard Protocol Items: Recommendations for Interventional Trials (SPIRIT) Figure includes the timeline for study enrollment, intervention, and assessment (Fig. 4). Study participants will be recruited and enrolled onsite at the 12 study sites. Staff of the five participating local organizations will work with study site staff to identify eligible participants. The site staff will create a master list of eligible ALHIV aged between 13 and 17 years who are enrolled in HIV care at their specific site. Parents and guardians of eligible participants will be briefly informed about the study, and after indicating interest, will be approached by trained ACT research assistants for written informed consent at routine clinic visits during the recruitment period. Research assistants will thoroughly explain all study procedures and obtain consent. Assent will be obtained from eligible 13- to 17-year-old ALHIV wards of consenting parents/guardians. Written informed consent will be obtained from eligible ALHIV under 18 years old who are deemed mature or emancipated minors and meet the following criteria: have been granted the status of adulthood by a court order; have lived independent of parental guidance for a minimum of 1 year; are married; are living on the streets; or are the head of their household [32]. It is expected that recruitment will be completed between 1 July and 1 December 2017 (Additional file 1).

### Data collection

After obtaining informed consent and/or assent, research assistants will collect sociodemographic and medical history information onto enrollment forms by face-to-face interview with study participants and/or their caregivers. Four psychosocial health questionnaires (described below) will also be completed at enrollment by study participants. Completion of the four questionnaires will be repeated at 12, 18, 24, and 36 months after enrollment, coinciding with the transfer time point, and 6, 12, and 24 months post transfer, respectively (Table 1). Blood viral-load sampling will be performed on the same schedule, and the results of all viral-load tests will be reported to clinic providers and filed in participants’ medical charts for use in HIV management. Per national treatment guidelines, stable patients who have been on HIV treatment for at least 6 months should have at least one viral-load test every year [31]. Clinic visits will also be tracked to measure the primary outcome of retention in care.

**Table 1:** Adolescent Coordinated Transition (ACT) study timeline, visits, and procedures

Visit	Timeline	Study visit	Procedure	Intervention group	Control group
Enrollment	0 months	Yes	Enrollment	Yes	Yes
			Readiness assessment	Yes	Yes
			Psychosocial assessment^a^	Yes	Yes
			Viral load	Yes	Yes
Visit 1	3 months	Yes	Type of clinic	Pediatric clinic	Pediatric clinic
			Organized support group	Yes	No
			Readiness assessment	Yes	Yes
Visit 2	6 months	Yes	Type of clinic	Adult clinic	Pediatric clinic
			Organized support group	Yes	No
			Readiness assessment	Yes	Yes
Visit 3	9 months	Yes	Type of clinic	Pediatric clinic	Pediatric clinic
			Organized support group	Yes	No
			Readiness assessment	Yes	Yes
Visit 4	12 months	Yes	Type of clinic	Adult clinic	Pediatric clinic
			Organized support group	Yes	No
			Readiness assessment	Yes	Yes
			Viral load	Yes	Yes
Transfer to adult care					
Visit 5	15 months	No	Type of clinic	Adult clinic	Adult clinic
			Organized support group	Yes	No
Visit 6	18 months	Yes	Type of clinic	Adult clinic	Adult clinic
			Organized support group	Yes	No
			Viral load	Yes	Yes
Visit 7	21 months	No	Type of clinic	Adult clinic	Adult clinic
			Organized support group	Yes	No
Visit 8	24 months	Yes	Readiness assessment	Yes	Yes
			Psychosocial Assessment^a^	Yes	Yes
			Viral load	Yes	Yes
End Visit	36 months	Yes	Type of clinic	Adult clinic	Adult clinic
			Readiness assessment	Yes	Yes
			Psychosocial Assessment^a^	Yes	Yes
			Viral load	Yes	Yes

^a^Psychosocial assessments performed with Health Locus of Control, Mental Health Continuum-Short Form, Functional Social Support Questionnaire and Sexual Risk Behavior, Beliefs, and Self-efficacy tools

In addition to collection towards evaluating the intervention’s effectiveness on primary and secondary outcomes, we will also collect data to describe and assess the implementation process, using the RE-AIM framework [28]. This will include: (1) Reach – the number and proportion of ALHIV willing to participate in ACT; (2) Adoption – the number and proportion of partner sites willing to implement the study; (3) Implementation – fidelity to the study protocol, including consistency of delivery of the alternating pediatric and adult clinic visits (PAPA); formation, curriculum adherence, and attendance at OSGs; presence and functionality of case management teams; and (4) Maintenance – the extent to which ACT becomes institutionalized at the intervention sites after completion of study follow-up [28].

### Outcome measures

The primary outcome measure is the *proportion of ALHIV retained in care at 12 and 24 months post transfer.* For this study, retention is defined as having made at least two clinic visits separated by a 6-month period within 12 months and at least four visits each separated by at least 6 months within 24 months post transfer. Hence, there are two primary outcome measures, with the long-term (24-month) retention measurement being a necessary “composite” of those who would have already demonstrated short-term (12-month) retention.

Secondary outcome measures are as follows:

1. *Proportion of ALHIV achieving viral suppression measured at 12 and 24 months post transfer*. Viral suppression is defined per the 2016 Nigerian national HIV guidelines as < 20 copies of HIV ribonucleic acid (RNA) per milliliter of blood plasma after at least 6 months of taking ART [33]
2. *Proportion of ALHIV demonstrating psychosocial wellbeing during study follow-up.* This study team has previously reported that poor psychosocial wellbeing and lack of adequate coping/self-efficacy skills are potential barriers to successful outcomes, including transitioning for this population in Nigeria [22, 34]. In order to generate more comprehensive evidence on wellbeing and resilience among ALHIV, we will administer four questionnaires among our study cohort: (1) the Health Locus of Control (HLC) scale will assess the extent to which ALHIV consider their health and health decisions to be within, or outside of their control; (2) the Mental Health Continuum-Short Form (MHC-SF) will measure the constructs of social, emotional, and psychological wellbeing; (3) the Functional Social Support Questionnaire (FSSQ) will evaluate the strength of the social support network of ALHIV; and (4) the psychosocial determinants of sexual risk behavior using the Sexual Risk Behavior, Beliefs, and Self-efficacy (SRBBS) scale will investigate for key psychosocial variables affecting ALHIV sexual risk-taking or protective behaviors [34–40]

The HLC tool has previously been validated by our research team among Nigerian ALHIV, and the MHC-SF has been validated among South African adolescents and adults [34, 40]. All the aforementioned tools will be administered by trained research assistants to study participants for a total of five times during the 3-year follow-up: at enrollment, transfer, and at 6, 12, and 24 months post transfer (Table 1). Additionally, healthcare transition readiness will be assessed twice (at enrollment and at transfer) using the Transition Readiness Assessment Questionnaire (TRAQ) [41, 42]. In a recent systematic review, the TRAQ was determined to be the best-validated transition tool with robust content and construct validity, as well as internal consistency [43].

### Sample size estimation

The design requires two arms (control = direct transfer without transition period or OSG and intervention = graduated transition with OSG). Six geopolitical zones are included in the design, with two sites (one replicate per arm) located in each zone. The factors considered for the sample size estimate are: at least 80% power, assuming 20% effect size, a confidence level of 95%, a correlation among repeated measures of 0.3, and an intra-class correlation within clusters of 0.05. Further, we assume equal numbers of clusters between CG and IG, and clusters within those conditions to be of equal size. Based on these assumptions, a baseline number of 15 participants per site was needed. This was, however, adjusted for several assumed losses. Based on knowledge of healthcare in this area and among target participants, the following participant loss estimates were made: healthcare transfers out of study sites at 5%, participant moving out of study area at 5%, deaths during the study at 7%, and study withdrawal/dropout at 5%. This results in a total of 22% dropouts, requiring an additional three participants (15 × 0.22) for a total of 18 participants per site. Additionally, we anticipate a 30% rate for declining to participate. Hence, we will need to approach an additional five ALHIV (18 × 0.3) for a total of 23 ALHIV currently enrolled as taking ART at study initiation to ensure that the requirement of 18 participants per site is met. Therefore, for two arms across six zones, we have a recruitment goal of 276 ALHIV (23 × 2 × 6) with a desired final sample size of 216 ALHIV (18 × 2 × 6) consented to participate. Sample size estimation was performed using G*Power (v. 3.1.9.1, Universität Düsseldorf, Germany) and Sampsize (v 0.6, P. Glaziou, ampsize.sourceforge.net) with appropriate adjustments for cluster randomization [44].

### Statistical analysis

For the primary objective, the study will assess the differential impact that the ACT intervention has on the primary outcome of retention. For this measure, ACT will test the difference in the percentage of study participants completing scheduled clinic visits under the hypothesis that the IG’s percentages will exceed CG percentages from baseline to 12 months (“short-term” retention) and to 24 months (“long-term” retention) following transfer to adult care. An initial test of proportions will be conducted at each time period to compare these percentages using a one-way test, and exact *p* values will be calculated using a permutation distribution. Additionally, a Poisson regression model with a time offset will be explored, which will allow for the development of a model to compare rates between the control and intervention arms while accounting for events (i.e., retention) per unit time, which allows time to vary for each subject as well as allowing the inclusion of potential covariates (for example, age, and time since HIV diagnosis).

Similarly, for secondary objective 1, the study will assess the differential impact that the intervention has on viral suppression. For this measure, ACT will test the difference in the percentage of study participants achieving viral suppression, under the hypothesis that the IG percentages will exceed CG percentages at each time period. The strategy in this analysis is similar to that of the primary objective: a one-way test of proportions will be conducted initially, followed by a Poisson count model to account for potential covariates of interest in addition to a temporal component.

For secondary objective 2, the study will assess whether the ACT intervention leads to improvements in the social, emotional, and psychological wellbeing of study participants; improvements in their self-efficacy; or changes in their health locus of control (HLC) from an external to an internal locus of control. Additionally, ACT aims to understanding the potential of psychologically based intervening variables to influence important study outcomes (i.e., increased retention rates and suppressed viral load). To examine important temporal changes in psychological factors, mean changes will be calculated for HLC, FSSQ, MHC-SF, and SRBBS scores from the transfer baseline to 12 and 24 months post transfer. Additionally, between-group comparisons will be made using repeated-measures analysis of variance (ANOVA). Owing to multiple tests among the same subjects, an approximate type 1 error of .05 will be maintained either directly through the use of a technique designed to accommodate repeated-measures designs (e.g., RM-ANOVA), or through the use of post-hoc adjustment of *p* values (e.g., Bonferroni’s method).

Simultaneously, ACT will examine direct and indirect contribution of psychosocial factors on viral-load suppression and retention of study participants in both study arms through multiple mediation model analyses as recommended by Preacher and Hayes [45]. Mediation analyses are particularly useful in randomized control trials as they attempt to capture the effects or processes that occur after randomization. As noted by Preacher and Hayes, the multiple mediation model is preferred over the traditional simple mediation model [45]. Additional mediator/moderator models will also be explored in order to investigate the potential direct and indirect impacts of other ACT intervention components; these will include the use of peer-led support groups during the pre- and post-transfer period, and the relative impact of case management support on retention rates and viral-load measures.

### Data management

Personal health information (PHI) and all study data will be kept confidential following the Health Insurance Portability and Accountability Act (HIPAA) and Nigeria’s National Health Research Ethics Committee guidelines. We will implement strategies at each stage to reduce the potential for breach in confidentiality and inadvertent disclosure of PHI. Collected information in paper form will be stored in a locked, fireproof cabinet in the Nigeria-based principal investigator’s (PI’s) office. Data will be abstracted and stored in an electronic database which will be encrypted and accessed by PIs, study coordinators and data management staff only. De-identified data will be made available to other members of the team, such as the biostatistician, on a need-to-know basis. Blood samples for viral load will be collected according to established protocol at each study site, and analyzed onsite or at an established referral laboratory with a de-identified code. The results will be available directly to the research team from the laboratory, and also filed in the clinic chart for access by medical providers.

### Data Monitoring Committee

The Data and Safety Monitoring Board (DSMB) will comprise four professionals in the research or HIV/adolescent care field and one layperson; a patient advocate from the study community.

The DSMB will perform the following functions:

- Review the research protocol and data and safety monitoring procedures, and evaluate the progress of the interventional trial
- Recommend halting the trial if it perceives that harm is occurring due to the intervention
- Meet with PIs at least once annually to review adverse event reports, participants’ complaints if any, and accrual
- Evaluate confidentiality and integrity of the database, and the procedure for storing confidential files
- Make recommendations to the study funder, relevant ethics committees, and investigators concerning continuation or conclusion of the trial

### Study staff recruitment and training

In the intervention arm, each participating healthcare facility will identify a staff member (nurse/midwife/social worker/expert patient) who will serve as the patient advocate on the site’s case management team. Along with the pediatric and adult HIV clinic focal persons, the patient advocate will form a case management team to coordinate care and support the ALHIV through the graduated transition. Peer facilitators may be selected by the healthcare team or nominated by ALHIV clients at the site, where appropriate. Central study coordinators and implementing partner-specific study coordinators from each of the five local ACT organizations have also been identified.

Training will be conducted in three phases:

1. A central training to orient focal persons, study coordinators, data manager, and support group staff to the study and to obtain feedback on study instruments
2. A second central training will be held to train research assistants on study protocols, ethical considerations, and data collection and management
3. Site-level trainings will be conducted for research assistants, peer facilitators, and other site-level research staff. Training will focus on study procedures including recruitment and enrollment, ethics, and OSG establishment and facilitation. Peer facilitator training will be based on four existing guides for organizing HIV support groups: “Positive Connections” [46], the INSPIRE MoMent Nigeria curriculum [47], Africaid’s Zvandiri curriculum [48], and the Malawi Teen Club Curriculum [49]

## Discussion

Nigeria is a country with a high HIV burden, with the second largest population of ALHIV worldwide. Therefore, successful interventions in Nigeria will have significant impact on health outcomes indicators among ALHIV in Africa and globally. Ineffective transfer from pediatric to adult care is associated with poor health outcomes. This can be addressed with comprehensive transition procedures that attend to the adolescent’s psychosocial needs, reducing the sense of loss at leaving the pediatric family, establishing trust in the adult care team, and providing peer support. Ultimately, higher post-transition retention in care, treatment adherence, and psychosocial wellbeing can be achieved for this population.

### Strengths

The ACT trial has several strengths. The study team’s Nigeria and U.S.-based researchers have extensive HIV research and program experience, a well-established local presence, and a track record of successfully conducting implementation research in Nigeria. The design is a cluster-randomized trial built on existing programs, which allows for determination of the real-life effectiveness of the intervention using triangulation of multiple concurrent data collection methods. Additionally, the ACT trial’s intervention encompasses the core elements of healthcare transitioning, including a standard transition procedure, transition readiness assessment, tracking, monitoring, and planning, and transfer of care. Lastly, the trial has strong support from local implementing partners that exert leverage on existing in-country PEPFAR investments, familiarity with local communities and the support of collaborating sites and government agencies.

### Potential limitations

There are some potential limitations in our study. First, HIV care at the 12 study sites is supported by five different NGOs, which might create some difficulties for coordination and quality control of study activities. To mitigate this potential problem, one NGO, IHVN, is charged with centrally coordinating and maintaining quality control for key study activities and data. Related to this, to ensure an acceptable level of quality of care for ALHIV, we also restricted eligibility to facilities with experience in and adequate staffing for HIV care delivery. However, this might reduce the generalizability of our findings for facilities with limited staffing. Lastly, our study population comprises adolescents who are likely mostly perinatally infected, which may limit the applicability of study findings among adolescents who acquired HIV through other routes of transmission.

### Potential implications for practice and research

Findings from the ACT trial will potentially contribute evidence towards addressing the high AIDS-related mortality rates observed among adolescents, especially in sub-Saharan Africa. Implementation research that utilizes the large PEPFAR and other funding investments and existing program infrastructure in resource-limited countries is more likely to be feasible, sustainable and expediently scalable. Finally, successful transition models for adolescents living with HIV can be adopted into research and/or practice for adolescents living with other chronic illnesses such as sickle cell anemia, asthma, and diabetes. Findings from this trial will be communicated through scientific publications, international and local conference presentations, and dissemination to policy-makers.

### Future research

Given the importance of cost-effectiveness in the selection and design of the study interventions, cost-effectiveness analysis for the ACT intervention is planned, especially where findings show significant positive impact on study outcomes.

### Trial status

Recruitment of participants for this study started the week of 26 June, 2017 and is expected to end on 1 December 2017. This trial was registered with ClinicalTrials.gov (NCT03152006) on 16 May 2017.

## Additional file

Additional file 1: SPIRIT 2013 Checklist: recommended items to address in a clinical trial protocol and related documents. (DOC 118 kb)

## Abbreviations

ACT: Adolescent Coordinated Transition
ALHIV: Adolescents living with HIV
APIN: APIN Public Health Initiatives Limited
ART: Antiretroviral therapy
CCCRN: Center for Clinical Care and Clinical Research
CIHP: Center for Integrated Health Programs
DSMB: Data Safety Monitoring Board
FSSQ: Functional Social Support Questionnaire
HIPAA: Health Insurance Portability and Accountability Act
HLC: Health Locus of Control
IHVN: Institute of Human Virology
LTFU: Loss to follow-up
MHC-SF: Mental Health Continuum-Short Form
NHREC: National Health Research Ethics Committee
NISA: Nigeria Implementation Science Alliance
OSG: Organized support group
PAPA: Pediatric-Adult-Pediatric-Adult
PEPFAR: President’s Emergency Plan for AIDS Relief
PHI: Personal health information
SCT: Social cognitive theory
TRAQ: Transition Readiness Assessment Questionnaire
