# Correction to: Adolescent Coordinated Transition (ACT) to improve health outcomes among young people living with HIV in Nigeria: study protocol for a randomized controlled trial

**DOI:** 10.1186/s13063-018-2463-4

**Published:** 2018-02-13

**Authors:** Nadia A. Sam-Agudu, Jennifer R. Pharr, Tamara Bruno, Chad L. Cross, Llewellyn J. Cornelius, Prosper Okonkwo, Bolanle Oyeledun, Hadiza Khamofu, Ayodotun Olutola, Salome Erekaha, William Nii Ayitey Menson, Echezona E. Ezeanolue

**Affiliations:** 1 Pediatric and Adolescent HIV Unit, Clinical Services, and International Research Center of Excellence, Institute of Human Virology Nigeria, Abuja, Nigeria; 2 Division of Epidemiology and Prevention, Institute of Human Virology, University of Maryland School of Medicine, Baltimore, OH USA; 3 Global Health Initiative, School of Community Health Sciences, University of Nevada Las Vegas, 4505 S. Maryland Parkway, Las Vegas, NV 89154 USA; 4 School of Medicine and School of Community Health Sciences, University of Nevada, Las Vegas, NV USA; 5 School of Social Work and College of Public Health, University of Georgia Athens, Athens, GA USA; 6 APIN Public Health Initiatives, Abuja, Nigeria; 7 Centre for Integrated Health Programs, Abuja, Nigeria; 8 FHI 360, Abuja, Nigeria; 9 Center for Clinical Care and Clinical Research Nigeria, Abuja, Nigeria

## Correction

In the original publication [1] the figure captions of Figs. 2 and 3 were reversed. The correct version can be found in this Erratum.

Incorrect version Fig. 2:

**Figure:**
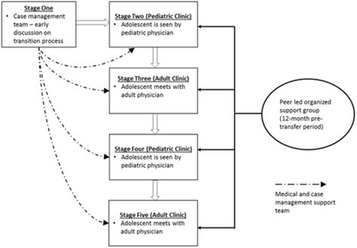
**Fig. 2** Consolidated Standards of Reporting Trials (CONSORT) flow diagram for Adolescent Coordinated Transition (ACT) trial site selection

Correct version Fig. 2:

**Figure:**
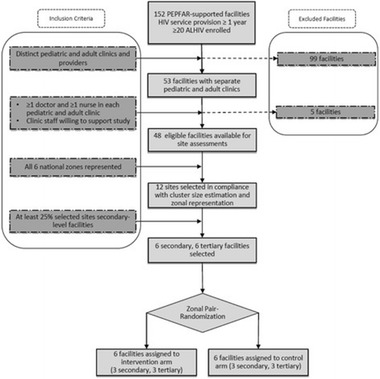
**Fig. 2** Adolescent Coordinated Transition (ACT) transitioning model showing the 12-month pre-transfer period (adapted from Maturo et al. [19])

Incorrect version Fig. 3:

**Figure:**
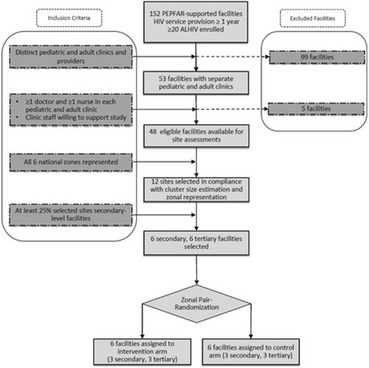
**Fig. 3** Adolescent Coordinated Transition (ACT) transitioning model showing the 12-month pre-transfer period (adapted from Maturo et al. [19])

Correct version of Fig. 3:

**Figure:**
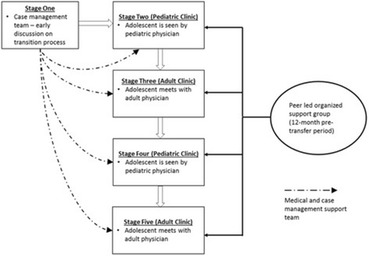
**Fig. 3** Consolidated Standards of Reporting Trials (CONSORT) flow diagram for Adolescent Coordinated Transition (ACT) trial site selection
